# Role of Non-Coding RNAs in the Progression of Liver Cancer: Evidence from Experimental Models

**DOI:** 10.3390/cancers11111652

**Published:** 2019-10-25

**Authors:** April O’Brien, Tianhao Zhou, Christopher Tan, Gianfranco Alpini, Shannon Glaser

**Affiliations:** 1 Department of Medical Physiology, College of Medicine, Texas A&M University, Bryan, TX 77807, USA; aprilobrien@tamu.edu (A.O.); tianhaozhou@tamu.edu (T.Z.); 2 Baylor Scott and White Health, Temple, TX 76508, USA; christopher.tan@bswhealth.org; 3 Richard L. Roudebush VA Medical Center, Indiana University School of Medicine, Indianapolis, IN 47405, USA; galpini@iu.edu; 4 Division of Gastroenterology and Hepatology, Department of Medicine, Indiana University School of Medicine, Indianapolis, IN 47405, USA

**Keywords:** liver cancer, hepatocellular carcinoma (HCC), noncoding RNAs, animal models

## Abstract

Liver cancer is a devastating cancer that ranges from relatively rare (around 2% of all cancers in the United States) to commonplace (up to 50% of cancers in underdeveloped countries). Depending upon the stage of pathogenesis, prognosis, or functional liver tissue present, transplantation or partial hepatectomy may be the only available treatment option. However, due to the rise in metabolic syndrome and the increasing demand for livers, patients often wait months or years for available organs. Due to this shortage, doctors must have other treatment options available. One promising area of cancer research lies in understanding the role of regulatory non-coding RNAs (ncRNAs) as oncogenic drivers and potential targets for prospective therapies. While the role of these ncRNAs was not initially clear, many of them have since been recognized to function as important players in the regulation of gene expression, epigenetic modification, and signal transduction in both normal and cancer cell cycles. Dysregulation of these different ncRNA subtypes has been implicated in the pathogenesis and progression of many major cancers including hepatocellular carcinoma. This review summarizes current findings on the roles noncoding RNAs play in the progression of liver cancer and the various animal models used in current research to elucidate those data.

## 1. Introduction

miRNAs, first discovered in 1993 by Lee and colleagues in the nematode *Caenorhabditis elegans* [1], are a category of ncRNAs that are 19–22nt in length and that function primarily by mediating post transcriptional gene silencing. Lee et al. initially found that in the nematode *Caenorhabditis elegans*, the downregulation of the protein *LIN-14* was essential in the progression from the nematode’s first larval stage [2]. Downregulation of *LIN-14* is dependent on the transcription of a second gene, *LIN-4*. It was later discovered that the transcriptional products of *LIN-4* had antisense complementarity to multiple sites in the 3′ untranslatable region (3′ UTR) of *LIN-14* mRNA [2,3]. The binding between these complementary regions decreased *LIN-14* protein expression, which allowed for the progression of *C. elegans* development. In summary, miRNAs are transcribed in the nucleus and form distinctive hairpin structures that can bind to their complementary messenger RNA targets in the cytoplasm. This usually happened at their 3′-untranslatable regions (3′ UTRs) or open reading frames, blocking the translation of proteins [4]. 

Small interfering RNAs of 21–23 nt also fall within the small noncoding RNA class. While they differ in their origin from miRNAs, they are similar in both size and function. Additionally, mi- and siRNAs share the Dicer and Ago family of proteins to release them from the precursor forms and participate in their silencing response. One notable difference between these similar RNA classes are their origins; siRNAs are often derived from the environment and can be thought of as defenders of the genome. Once a part of the RNA-induced silencing complex, or RISC complex, these siRNAs will guide the RISC to their complementary targets for degradation [5]. 

Another small ncRNA is the piRNA (PIWI interacting) class, commonly referred to as PIWI RNAs. These 23–36 nt long ncRNAs were first discovered by Aravin et al. [6] during the profiling of small RNAs during *Drosophila* development. First described as repeat-associated siRNAs, they were eventually named for their ability to form complexes with the P-element Induced Wimpy (PIWI) proteins of the Argonaute family, where they fit into specific binding pockets and serve as guides to lead the Argonaute protein to its target mRNA [7]. After their initial discovery in *Drosophila*, piRNA classes were found to be abundantly present in the gonads of various vertebrates and invertebrates [8], and were determined to play a key role in the suppression of transposon activity during germ line development [9]. However, growing evidence suggests that the piRNA pathway genes extend into somatic cells as well [10]. In germ cells, the synthesis of piRNAs occurs in an electron-dense perinuclear area of the cell, whereas in somatic cells, the processing of these molecules occurs in the outer membrane of the mitochondria [1]. The majority of these piRNAs exist as cluster loci that can be found in intergenic and genic regions [11]. There are three types of piRNA clusters: unistrand, dual strand and bidirectional. The unistrand cluster is the source of piRNAs that are generated from a single DNA strand. The dual strand cluster is the source that is generated from both DNA strands at a single gene location. Lastly, the bidirectional cluster generates piRNAs from both DNA strands, but transcription of these molecules occurs in opposite directions [1].

Somewhere between the classification of small and long noncoding RNA lies a unique set of RNA known as small nucleolar RNA. Despite their name, snoRNAs range in size from 60–120 nt C/D box snoRNAs to the larger (up to 300 nt) H/ACA box snoRNAs. Similarly to their smaller counterparts, snoRNA complexes have been found to be associated with the Argonaute family of proteins [12]. snoRNAs are best known for their role in the guided methylation of RNAs, but other novel roles for them have been described, such as functioning as miRNA post-modification [13].

The remainder of the ncRNAs are longer than 200 nt and are classified as long non-coding RNAs (lncRNA) [14]. As of 2015, advancements in microarray and gene sequencing have allowed for the identification of 58,648 lncRNA [15]. Like mRNAs, most lncRNAs are transcribed by RNA polymerase II. It has also been found that these transcripts are able to be spliced, polyadenylated, and capped much like mRNAs, but they do not code for any protein [16]. lncRNAs are classified into five types based on their genomic location: sense, antisense, bidirectional, intronic, and intergenic. Sense lncRNAs overlap with the sense strand of a protein coding gene. Antisense lncRNA overlap with the antisense strand of a protein coding gene. When the sequence is oriented head to head with a protein coding gene, this is referred to as bidirectional. Intronic sequences are located inside the intron of a protein coding gene. Lastly, intergenic sequences are found in a region between two protein coding genes [1]. The functions of lncRNA are broad. They have been shown to play roles in the regulation of transcription, mRNA translation, nuclear structure organization, and certain protein activities [1]. There are several different mechanisms by which lncRNAs regulate the transcriptional process. It has been shown that they can directly interfere with promoter sequences [17] on their own, or do so by demethylating the promoter genes [18]. lncRNAs may also serve as coactivators of certain transcription factors [19] or block the binding of transcription factors to DNA [20]. While the various noncoding RNAs play unique roles in organisms’ development and function, their roles in cancer progression often overlap with individual members of the same RNA class, stimulating opposing pathways (Table 1). 

## 2. MicroRNAs

**Figure 1:**
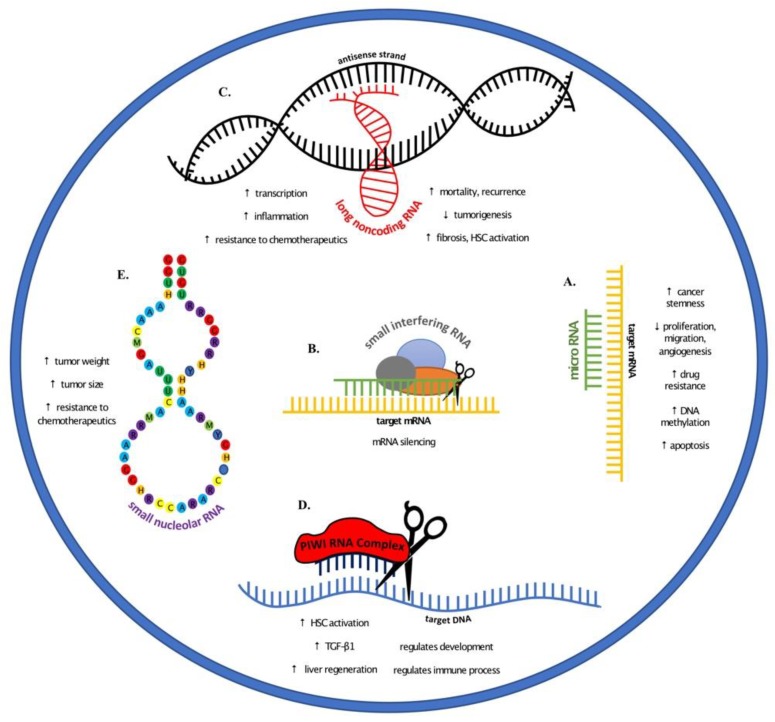
Noncoding RNAs and their roles in liver cancer elucidated through various animal models. (**A**) microRNAs regulate cancer progression through increasing stemness, drug resistance, and DNA methylation. Conversely, microRNAs play a role in decreased proliferation, migration, and angiogenesis, and increased apoptosis. (**B**) Small interfering RNAs target messenger RNA and silence their transcription. (**C**) The role of long noncoding RNA (lncRNA) varies with increased transcription, inflammation, resistance to chemotherapeutics, and fibrosis. In addition to increased HSC activation, there are higher incidence of mortality and cancer recurrence. Other lncRNAs have also been indicated in decreased tumorigenesis. (**D**) PIWI RNA have been shown to activate HSCs and increase TGF-β1 expression and liver regeneration, in addition to regulating immune processes and development. (**E**) Small nucleolar RNAs provide resistance to chemotherapeutic drugs and have been shown to increase tumor size and weight.

MicroRNAs (miRNAs) have been identified as a valuable source of biomarkers for a wide range of pathological conditions across many organs, including liver cancer. Within the human genome, there may be an excess of 2000 mature miRNAs, which can regulate at least one-third of all human protein-coding genes [61,62]. MiRNAs are small noncoding RNAs (18–22 nucleotides) that regulate post-transcriptional gene expression through their binding to complementary 3′ untranslated regions (3′-UTRs) of their messenger RNA (mRNA) targets [63]. The biogenesis of miRNAs takes place via a multistep process. Primary miRNA (pri-miRNA) structures are initially transcribed in the nucleus by RNA polymerase II. These precursor structures are subsequently cleaved into pre-miRNAs by the DGCR8–Dorsha complex and translocated to the cytoplasm by Exportin 5 [64]. The pre-miRNAs are then processed into duplex RNAs (about 22 nucleotides) by the endoribonuclease Dicer, and mature single-strand miRNAs are produced by association with the RNA-inducing silencing complex (RISC). Within the RISC, mature miRNAs can bind to target mRNAs and repress the translation of mRNAs into proteins by preventing their translation and mRNA degradation [65,66]. Deregulation of miRNA biogenesis is commonly observed in liver cancer. Profiling studies from different groups have reached similar conclusions that miRNA expression can be distinguished between liver cancer patients and normal healthy individuals [67,68,69]. Interestingly, dysregulated miRNAs can have different functional roles in cancer development through various cellular mechanisms such as cell proliferation, cell cycle regulation, cell migration and invasion, apoptosis, etc. (Figure 1A). While some miRNAs promote carcinogenesis by downregulation of tumor suppressor genes or activation of oncogenes, some miRNAs have been implicated to do the opposite [21]. 

### 2.1. Xenograft Models

Xenograft models have been widely used in cancer research, and can be established either by direct implant of tumor tissue fragments or by injection of liver tumor cell lines into recipient mice. Most implantation studies in HCC are performed with a subcutaneous xenograft model, in which human HCC cells are implanted subcutaneously (usually in the flanks) into immunodeficient mice, including nude mice, severe combined immune deficient (SCID) mice, or non-obese diabetic-severe combined immunodeficiency disease (NOD/SCID) mice [22]. For example, Fang et al. injected HCC cells stably expressing miR-124 into the flanks of nude mice to evaluate the potential role of miR-124 in the pathogenesis of HCC. With the ectopic overexpression of miR-124, the tumor sizes and the number of intrahepatic and pulmonary metastatic nodules were significantly decreased, which may have been associated with two miR-124 target genes (*ROCK2* and *EZH2*) [23]. Besides miR-124, miR-151 and the miR-200 family have also been shown to be downregulated in subcutaneous xenograft models of HCC, and to negatively regulate different components of the RHO/RHO-associated protein kinase (ROCK) pathway to inhibit cancer cell invasion and metastasis [28,70]. As cancer stem cells play important roles in the progression of HCC growth, Meng et al. used SCID mice with subcutaneously injection of human hepatocellular cancer stem cells (HSCs) to study the functional role of miRNAs in cancer stem cells. The authors described that inhibition of *Twist*/miR-181 led to reduced HSCs invasion and metastasis, whereas silencing of *IL-6*/let-7 increases the chemosensitivity of HSCs to sorafenib and doxorubincin [24]. In addition, microRNA deregulation is not only limited to HCC, but can also be found in the other primary source of liver cancer, cholangiocarcinoma [71]. Han et al. conducted a circadian rhythm study in CCA by subcutaneous injection of CCA cells (stably transfected with *Per1*) into nude mice. *Per1* is a key circadian clock gene that is regulated by miR-34a. miR-34a-dependent overexpression of *Per1* reduced tumorigenesis of CCA and the expression of other clock-controlled genes [72]. Another CCA study evaluated the effect of a miR-24 inhibitor in a xenograft mice model subcutaneously injected with the inhibitor-transfected CCA cells. Knocking down miR-24 negatively regulated *menin* expression and decreased CCA cell proliferation, migration, and angiogenesis [73].

Besides the subcutaneous xenograft models, orthotopic implantation of tumor cells or tumor tissue fragments has also been widely used to study liver cancer. Wong et al. used this approach to evaluate the role of miR-139 in HCC metastasis. miR-139-expressing HCC cells were first injected into nude mice to induce development of subcutaneous tumors. Cubes of the tumors (1 mm^3^) were cut and orthotopically implanted in the left hepatic lobe of the nude mice. Overexpression of miR-139 suppressed metastasis and progression of HCC by down-regulating *ROCK2* [29]. In addition, the other common approach to orthotopic implantation is to suspend tumor cells in medium containing 50% Matrigel and directly inject the Matrigel into the liver of the mouse [74]. miR-130b was shown to promote cancer stemness properties by enhancing chemoresistance, tumorigenicity, and self-renewal in orthotopic xenograft SCID mice [32]. Furthermore, recent findings have defined a role for miR-21 in patient-derived organoids (PDOs). Lampis et al. created organoids from patient biopsies to determine their mutations and architecture. Utilizing both in vivo and in vitro systems, the researchers found an inverse correlation with the HSP90 inhibitors and the baseline levels of miR-21. miR21 appeared to act through the HSP40 family member B5, and the transgenic expression of this molecule in CCA cell lines reintroduced HSP90 inhibition sensitivity. This data provided insight into the miR21-specific weakness in CCA, and proof-of-concept for the creation of organoids from patient biopsies in order to provide more specificity in patient treatments in cholangiocarcinoma [33]. 

### 2.2. Chemical-Based Models

Diethylnitrosamine (DEN) is a carcinogenic compound which is frequently used to experimentally induce liver fibrosis and/or HCC in animals. The incidence of DEN-induced HCC is determined, at least in part, by the strain. C3H and B6C3F1 mice are more likely to develop HCC compared to C57BL mice [34]. Recent studies have demonstrated miR-221 to be an oncogenic miRNA which plays an important role in the progression of HCC. For example, miR-221 overexpression is associated with sorafenib resistance in a DEN-induced HCC rat model, presumably by modulating caspase 3 expression and cellular apoptosis [40]. In another study, treatment with an antisense 2-O-methyl oligoribonucleotide targeting miR-221 reduced tumor growth in miR-221tg-DEN-induced HCC mouse models [41]. In addition, Wu et al. explored the concurrent increase of liver progenitor cells (LPCs) and transforming growth factor-β (TGF-β) in DEN-induced rat hepatocarcinogenesis. They demonstrated that TGF-β-induced Akt activation and LPC transformation was mediated by miR-216a-modulated PTEN suppression [35].

Carbon tetrachloride (CCl_4_) is another hepatotoxin widely used to induce hepatocarcinogenesis in rodent animals. In a study with 14 week CCl_4_ treatment, anti-miR-221 oligonucleotides or miR-199a-3p mimics prevented HCC progression through the control of multiple cancer-associated molecular pathways, which included PI3K–AKT, WNT-β-catenin, cell cycle, invasion, and motility. However, there were no apparent effects on liver fibrosis and cirrhosis, which was suggested by the similar expression of α-SMA between treatment groups [46]. miRNA profiling analysis from liver tissues treated with CCl_4_ or corn oil for 10 weeks showed that hepatic expression of miR-378 family members (miR-378a-3p, miR-378b, and miR-378d) was decreased in CCl_4_-treated mice compared with the control group, which was inversely correlated with the *Hedgehog* target genes, *Gli2*, and *Gli3* in tumor and non-tumor tissues [47]. Interestingly, Marrone et al. evaluated the role of miRNAs in the pathogenesis of hepatocarcinogenesis in male B6C3F1/J mice treated with DEN or CCl_4_ alone, or a combination of DEN and CCl_4_. Different tumor incidences were observed in the following order: DEN < CCl_4_ < DEN + CCl_4_ [48].

### 2.3. Genetically Modified Models

Hepatitis B virus (HBV) is a well-recognized risk factor for HCC. Many types of transgenic mice harboring hepatitis B virus X (HBx) protein and hepatitis B virus surface antigen (HBsAg) have been generated. Wang et al. generated *HBx* and *HBsAg* knockin transgenic mice by integrating these two genes into the same *p21* locus. Both male and female HBx transgenic mice developed HCC after the age of 18 months, whereas only male *p21*–*HBsAg* transgenic mice between the ages of 15 and 24 months developed HCCs, suggesting that men might be more susceptible to HBV-induced HCC than women [49]. Using the same *p21–HBx* transgenic mice, Zhang et al. showed that local liver metastasis and distant lung metastasis were significantly inhibited by blocking miR-143. Upregulation of miR-143 expression, transcribed by NF-kB, promotes cancer cell invasion/migration and tumor metastasis by repression of *FNDC3B* expression [75]. In addition, Huang et al. showed that miR-152 was downregulated in the livers of *HBx* transgenic mice. Downregulation of miR-152 led to enhanced DNA methylation with upregulation of *DNMT1*, which further promoted HCC development [58]. Autophagy pathway was suppressed and inversely correlated with miR-224 expression in liver tumors of *HBx* transgenic mice [76]. 

Hepatitis C virus (HCV) is another major cause of HCC worldwide. Transgenic mice containing the complete core gen of HCV were generated by Koike et al. to study the role of HCV core protein in HCC [50]. Surprisingly, no study has been conducted to examine the role of miRNAs in HCV-induced-HCC transgenic models. In a HCV infection-induced mice model, miR-155 has been identified to promote cell proliferation and HCC tumor growth by increasing Wnt/β-catenin signaling [51]. 

*Multidrug resistance-associated protein 2* (*Mdr2*) knockout mice have been extensively studied over the decades, as they provided a model for studying cholestasis, biliary fibrosis, and hepatic carcinogenesis. Livers of 3 month old *Mdr2^−/−^* mice had characteristic portal inflammation and bridging fibrosis, which was followed by hepatocarcinogenesis at 15 months with nearly 100% efficiency [52]. Wu et al. showed that miR-200b expression was increased in *Mdr2^−/−^* mice and decreased in *Mdr2^−/−^* mice subjected to dark exposure or melatonin treatment. Inhibition of miR-200b in *Mdr2^−/−^* suppressed biliary proliferation, liver fibrosis, and angiogenesis [55]. In addition, as extracellular vesicles (EVs) containing miRNAs can play important roles in various liver diseases, liver-stem-cell-derived EVs (LSCEVs) and human-hepatocyte-derived EVs (HHEVs) were injected into *Mdr2^−/−^* mice to evaluate miRNA profiling and their downstream target genes [56]. Compared to HHEVs, LSCEV-injected mice showed increased levels of several miRNAs, including let-7a, let-7b, and miR-25. LSCEVs reduced ductular reaction and biliary fibrosis in *Mdr2^−/−^* mice through let-7-dependent reduction of *Lin28a*, *Lin28b*, *IL-13*, *NF-κB*, and *NR1H4*, and enhancement of *FoxA2* [57].

Tetracycline-controlled systems have been widely used for temporal control over genetic changes. Shachaf et al. crossed *TRE–myc11* mice with a transgenic line, *LAP–tTA10*, where the liver activator protein (LAP) promoter drives expression of the tetracycline trans-activating protein (tTA) in liver cells. In this tet-o-myc mouse model, overexpression of *myc* in adult mice can reproducibly induce HCC (PMID: 15475948). Using the same tet-o-myc transgenic mouse model, Kota et al. showed that miR-26a could specifically reduce cancer cell proliferation, promote tumor-specific apoptosis, and suppress hepatocarcinogenesis [36]. 

Genetic depletion of specific miRNAs is another animal model commonly used to study HCC. Hsu et al. evaluated the biological function of miR-122 through the generation and characterization of mice with liver-specific (LKO) and germline (KO) deletion of this miRNA locus [59]. Although a tumor suppressor role for miR-122 has previously been proposed based on in vitro studies and human HCC samples, these findings provided the first in vivo evidence that loss of miR122 is sufficient to initiate highly penetrant HCC development. They demonstrated that chronic loss of miR-122 caused steatohepatitis and altered liver function that eventually led to HCC [59]. Using the same miR-122 knockout mouse model, Valdmanis et al. observed that depletion of miR-122 increased miR-122 mRNA target expression and reduced the targets of other microRNAs, such as mRNA targets of *let-7* and miR-21 (*Nr6a1*, *Pdcd4*, *Srsf7,* and *Stag2*), suggesting that when one common miRNA is diminished, other microRNAs would utilize the newly available miRNA machinery to exert a greater repressive effect on their targets [77]. In addition, miR-148 deficiency mice significantly enhanced DEN-induced hepatocarcinogenesis through increased lipid metabolic disorders in mice, suggesting miR-148a as a potential therapeutic target for the regulation of hepatocarcinogenesis [25].

### 2.4. Other Models

Besides rodent animal models, miRNA research related to liver cancer has also been performed in mammalian animals. One recent study designed and validated a platform for ultrasound-mediated delivery of anticancer miRNA-loaded nanoparticles to pig liver and kidney [37]. In order to achieve controlled and sustained release of miRNAs into tumor cells, antisense-miR-21 and antisense-miR-10b have been encapsulated into nanoparticles and delivered guided by focused ultrasound in a noninvasive way. Compared to untreated control regions, miRNA expression increased 1.9- to 3.7-fold with the treatment of ultrasound, suggesting that this novel platform has therapeutic potential to improve the delivery efficiency of miRNA to target regions in liver and other organs in large animal models.

## 3. Small Interfering RNAs

Unlike microRNAs, small interfering RNAs are exogenous to the cell and defend the integrity of the organism’s genome. Beginning as long double-stranded RNAs, Dicer chops them into smaller nucleotides with overhanging ends. These, when removed from their latent strand, can then go on to silence target messenger RNA [5]. siRNAs not only work within the cell to silence the mRNA before it its message can be translated into protein (Figure 1B), they also provide an extremely useful genetic tool for scientists to manipulate pathways, thus determining the benefits and consequences of such manipulation. 

### 3.1. Xenograft Models

Due to the occurrence of controlled and rapid cell growth common to cancers, the pathways that regulate the cell cycle offer themselves as attractive targets in research. CDC25 phosphatases are one such target. *CDC25B* has been shown to be overexpressed in patient populations with hepatocellular carcinoma, and this is frequently associated with poor prognosis. Additionally, research has shown that *CDC25B* promotes cellular migration and invasion in HCC. Using a xenograft mouse model and siRNA oligos designed to deplete the *CDC25B* expression in Hep3B and Hep40 HCC cell lines, researchers then observed the growth and invasion over a 4 day time period. A group of 4 to 6 week old nude mice were injected with transfected cells every 2 days, and growth curves and tumor dimensions were plotted after a 50 day incubation period. Significance between the siRNA22–*CDC25B* group and the RNAiMAX was achieved by day 7, with P falling below 0.01 from day 15 onwards. In vitro data further supported these findings by inhibiting HCC cellular invasion and migration in the *CDC25B*-siRNA-treated cells. Altogether, these data support the rationale for specific small molecular inhibitors of *CDC25B* [38]. 

### 3.2. Chemical-Based Models

However, targeting the cell cycle of hepatocytes is not without complications, as this affects the overall health and regenerative ability of the liver. Exciting new data have been reported using siRNAs against ANLN, a cytoskeletal protein that is part of the scaffolding complex and participates in the regulation of cytokinesis and tumorigenesis. Human data showed an increase in ANLN mRNA in HCC patient samples when compared to the surrounding healthy liver tissue. Using models of both carbon tetrachloride and diethylnitrosamine, Zhang et al. injected ANLN knockdown H2.35 liver cells (FRG mice) or siRNA into LAP–MYC mice to demonstrate the role ANLN plays in survival and regeneration [75].

### 3.3. Genetically Modified Models

The researchers also developed mice with transgenes for siRNA against the mRNA of ANLN to determine the level of tumorigenesis in both DEN and CCl_4_ HCC models. All experimental models of hepatocellular carcinoma showed an overall decrease in the presence and size of liver tumors in both DEN- and CCl_4_-induced liver damage. However, despite the reduction in the tumors’ ability to grow, the hepatocytes did not lose their regenerative abilities and there was no difference in liver-to-body-weight ratios or increases in aspartase or alanine aminotransferase when compared to their control counterparts. Altogether, these data offer promising targets for treatments while taking into consideration the surgical resection many patients undergo [78].

Tumor suppressor protein, p53, plays a critical role in cell cycle regulation, differentiation, and apoptosis. One regulator of p53 is *E3 ubiquitin ligase MDM2*. This gene is often over-expressed in human tumors, and regulators of *MDM2* may provide valuable targets in the fight against liver cancer. Using the genetically modified *KLF^+/−^* and *KLF^+/+^* mouse models exposed to the chemical carcinogen DEN, Tarocchi et al. studied the impact on KLF6 loss and resultant tumor formations. Upon comparison of the tumor to liver surface ratio, the homozygous KLF animals had a dramatic decrease in the overall percentage when compared to their heterozygous KLF animal counterparts. By 9 months of age, the heterozygous animals had nearly twice the number of macro- and microscopic lesions. These data correlated with an increase in MDM2 and a corresponding decrease in the presence of p53. These findings provide additional information about the molecular abnormalities in HCC [30].

## 4. PIWI RNAs

Unlike long noncoding RNA‘s, PIWI-interacting RNAs are a class of silencing RNA molecules that are a mere 21–35 nucleotides in length. Their primary job is to guide PIWI proteins to methylate DNA and cleave target DNA. Through these mechanisms they are able to fight viral infections and regulate gene expression (Figure 1D), and they are most active in germ cell lines and cancer cells [31]. Due to the fact the PIWI RNAs have not enjoyed the extensive amount of research some of their noncoding counterparts have, they offer an opportunity for novel discoveries and pathway mapping in the ongoing fight against liver cancer. 

### 4.1. Chemical-Based Models

Although this research area is new, recent data has shown a role for piR-823 in the upregulation of *TGF-β1* during liver fibrosis. Utilizing the carbon tetrachloride mouse model of liver fibrosis, in addition to bile duct ligation (BDL), the researchers isolated the primary HSCs and collected total livers. Not only did the researchers show a correlation between piR-823 and HSC activation in both fibrotic models, they also presented data to suggest that piR-823 not only activated HSCs, its increased synthesis was also the result of activated HSCs, creating a vicious cycle. In addition to these observations, the researchers also noted an increase in *TGF-β1* in the HSCs treated with a piR-823 mimic. Providing a piR-823 antogomir reduced the *TGF-β1* levels further, supporting a role for this PIWI RNA in the progression of liver cancer [42]. 

### 4.2. Other Models

Often, the only treatment option for liver cancer is surgical resection. Research using the partial hepatectomy (PH) rat model has described a role for PIWI RNA in the proliferative response the liver undergoes following PH. First, the researchers determined if known piRNAs were present in normal rat liver tissue. The researchers found that around 1% of the small RNA molecules could be identified as piRNAs. Furthermore, the activity of several of the PIWIs was most prevalent at 24 and 48 hours, with a cessation at 168 hours post PH, corresponding with DNA replication and the activation of the cell cycle [43]. In corroboration of the aforementioned research, the research group of Dr Lv in Hangzhou, China, identified four novel piRNAs after performing partial hepatectomy on white spotted bamboo sharks. A one-third PH was performed on the right side of the liver, the sharks were recovered and continuously fed, and then an additional section was resected 3 hours later to look for upregulation of known and novel piRNAs. The researchers were able to identify six known and four novel piRNAs in normal liver tissue. Using a GO and KEGG pathway analysis, they were able to speculate that pi-1738 has a role in liver regeneration and pi-6084 regulates development and immune processes. These findings may provide therapeutic pathways and further understanding into the regenerative properties of the liver [44]. 

## 5. Small Nucleolar RNAs

Another group of small non-coding RNA, shown to play a key role in the pathogenesis of hepatocellular carcinoma, are the small nucleolar RNAs (Figure 1E). These RNAs are 60–300 nucleotides in length and can be divided into two major classes, the H + ACA snoRNAs and the C + D snoRNAs. They serve primarily to guide RNAs through post-translational synthesis, working in conjunction with additional proteins to form snoRNPs [60].

### Xenograft Models

Despite their role in methylation and post-translational synthesis, evidence has also emerged that describes a role for snoRNAs in human malignancies. SNORD50A and -B are often deleted in various cancers, while SNORA42 is often overexpressed in colorectal and lung cancers. Like SNORA42, *ACA11* is also overexpressed in colorectal cancer but lacked a definitive role is HCC. After demonstrating that *ACA11* was highly expressed in HCC tissue when compared to surrounding non-tumor liver tissue, the researchers utilized male BALB/c nude mice to inject stably transfected *ACA11*-knocked-down HCCLM9 cells into the animals’ right flank. Huh7 cells overexpressing *ACA11* were injected into the armpits and tissues were collected at 5 weeks. Overexpression of *ACA11* significantly increased tumor weight and growth when compared to the control tumors. In the knockdown cells the tumor weights were lower and there was a significant decrease in the *Ki67*^+^ hepatocyte nuclei compared to controls. The researchers then postulated that ACA11 was acting upon the PI3K/AKT pathway. Subsequent inhibition of this pathway reduced the stimulatory effects and the researchers concluded that there is biological significance of *ACA11* in HCC, and that further research may provide therapeutic targets [79]. However, the intricacies of cancer often mean that a single molecule, an individual target, or a specific pathway are a single strand in a complex web of pathogenesis. Additional research into the PI3K/AKT pathway yielded yet another snoRNA, SNORD126. Data from a microarray showed SNORD126 up- or downregulated a total of 500 separate genes. Due to the microarray results highlighting a link between SNORD126 and upstream regulators of *PI3K*, and the well-established role of dysregulation of the PI3K/AKT pathway in cancer progression, the researchers focused their attention there. Female nude mice were inoculated with either Huh-7 or SW480 SNORD126-expressing cells, or EGFP-expressing cells. These cell lines were chosen due to their low endogenous SNORD126 levels. Overexpression of SNORD126 increased levels of upstream receptors in the *PI3K* pathway, including *FGFR2* and *LPAR1*. Chemo-resistance was inferred by overexpression of SNORD126. Subsequently, downregulation decreased cell growth, suggesting a potential therapeutic pathway for cancer treatment [39].

## 6. Long Noncoding RNAs

One novel area of interest in the progression of liver cancer is long non-coding RNAs (lncRNA). lncRNAs are RNAs that comprise more than 200 nucleotides. Within the human genome, there may be in excess of 15,000 lncRNA and, similarly to mRNAs, their expression is highly regulated through transcription factors and methylated lysines [80]. Some predominant roles these lncRNAs play in liver cancer progression involve the renewal of the stem cells, cancer cell proliferation, communication, and even metastases (Figure 1C). 

### 6.1. Xenograft Models

Recent studies have indicated a clear role for lncRNA is the progression of liver cancer through the upregulation of lncARSR. In a tumor model utilizing NOC–SCID mice and hepatoma cells, serially diluted and mixed with either si-lncARSR or si-NC, there was a significant reduction in the number of tumors and an overall decrease in the cancer cell markers *CD133^+^* and *EpCAM* [17]. Additionally, inhibition of lncARSR impaired STAT3 activity, which has been previously shown to be an active participant in tumorigenesis [81]. Additional studies analyzing human samples and including the manipulation of another long noncoding RNA, DILC, supported the previous researchers′ assertion that STAT3 was involved in the negative regulation of hepatocellular carcinoma. However, the pathway targeted by the lncRNA DILC is specific to the *IL-6* promoter region, and a subsequent knockdown of lncDILC inversely affected the amount of EpCAM and CD24. NOD–SCID mice inoculated with the hepatoma cells isolated from mirDILC spheroids or mirCtrl spheroids had significant increases in both weight and volume. These findings were further validated by patient findings that correlated low levels of lncDILC expression with early recurrence and high mortality [53]. 

### 6.2. Chemical-Based Models

NOC–SCID mice are not the only animal models that have been shown to implicate a role for lncRNA in liver cancer progression. Liver fibrosis induced by carbon tetrachloride (CCl_4_) has been reported to be a result of dysregulation in multiple long non-coding RNA′s such as lncRNA-*COX-2*. Research utilizing the CCl_4_ mouse model of liver fibrosis at 2 and 3 months showed an upregulation in lncRNA-*COX-2* in cirrhotic areas of liver tissue compared to the control group. However, despite a significant increase in the CCl_4_ groups over the control, there was no difference noted between the 2 and 3 month CCl_4_ groups compared to one another [54]. *COX-2* has previously been implicated in both the growth of tumors and the evasion of the immune system, and this research supported a significant correlation with the RNA expression of *COX-2* and the level of liver fibrosis. These findings offer a therapeutic pathway to be explored. Another therapeutic pathway recently discovered in the carbon tetrachloride mouse model that offers promise in the progression of liver disease is the lncRNA nuclear paraspeckle assembly transcript 1 (*NEAT1*) discovered in primary mouse hepatic stellate cells (HSCs). In both the in vitro and in vivo models, there was a consistent and continuous increase in the relative expression of *NEAT1*. Adenoviral vectors against *NEAT1* significantly decreased the presence of collagen I fibers in liver tissue, among other markers such as *KLF6*. Regulatory microRNA-122 was found to inversely affect the expression of *NEAT1*, with significant increases occurring with the introduction of a miR-122 inhibitor [82]. The rat model CCl_4_ yielded similar results with regards to lncRNA-*GAS5* and its interaction with miR-23a through the PTEN/P13K/Akt pathway. Building upon their previous research implicating the upregulation of miR-23a and poor patient prognosis [5], they then focused on the role of lncRNA-*GAS5*. lncRNA-*GAS5* was identified through bioinformatics software and further confirmed using a luciferase vector. These promising data showed that the direct interaction between miR-23a and lncRNA-*GAS5* was an inverse relationship, with the result being an alleviation of hepatic fibrosis and reduction in the activation of HSCs [45].

Recent research using the diethylnitrosamine (DEN)-prompted HCC rat model evaluated a role for the lncRNA-*ecCEBPA* and -*UCA1*. lncRNA-*UCA1* has been previously implicated in various cancers [11,44,45] including gastric, lung, and prostate. It has also shown to increase cancers’ resistance to chemotherapeutics [83]. Along the lines of similar studies, *UCA1* was overexpressed in the DEN cancer model, with a transcription level increase of 650% compared to controls. In addition to *UCA1*, the lncRNA-*ecCEBPA* gene was also upregulated in the DEN-treated animals. These levels were decreased by treatment with S-adenosyl methionine (SAM) via the inhibition of the P13K and Akt signaling pathway [84]. 

### 6.3. Genetically Modified Models

The presence or overexpression of previously identified long noncoding RNAs in liver cancer do not necessarily yield the desired targets for therapeutics, but may offer biomarkers for liver carcinogenesis. A recent study supported this idea through the study of non-coding *nuclear-enriched abundant transcript 2* (*NEAT2*), commonly known as *Malat1*. Previous studies have shown that *Malat1*, expressed in high levels, correlates with poor prognosis in various cancers, including liver cancer [26]. Based upon this, and similar studies, the researchers generated a *Malat1* knockout mouse through the use of a gene trapping technique. However, despite the decrease in hepatic mRNA levels of *Ki67*, *TERT*, *Foxo3* and others, the overall tumorigenicity did not change when compared to the wild type controls [27]. Another popular model of liver disease is the *multidrug resistant 2* knockout mouse model of hepatocellular carcinoma. The benefit to the *MDR2* KO model is the dysregulation of several pathways that create a disease state that mimics the familial intrahepatic cholestasis found in humans. As a result, these animals provide a link between inflammation and HCC [85]. *MDR2* KO models may also be used to study the gender variations of cholestatic injuries. One such study explored the pronounced hepatobiliary progression of females over age-matched males through the exploration of lncRNA-*H19*. lncRNA-*H19* is an estrogen-targeted gene, maternally expressed and present in both murine and human cirrhotic livers. Data revealed that H19 levels were very low in the FVB control mice, with no significance noted between the male and female animals. However, the same time point in the disease model showed a 200-fold increase in the females’ expression of lncRNA-*H19*. Furthermore, the upregulation of *H19* was a result of the taurocholic acid activation of *S1PR2*, which was found to also be upregulated in human PSC patients, especially females. These very important data provide a focal point for further research into the gender disparity between severity of hepatobiliary injuries in men and women [86].

## 7. Conclusions

Currently, there are numerous well-established animal models in use in the study of liver cancer. These cover a wide range of targets and pathways, from genetically engineered knockin or knockout mouse models utilized to elucidate specific molecular roles, to bamboo sharks used in the study of the immune processes of the liver. Recent scientific discoveries in the area of noncoding RNAs have created a landscape of opportunity in pathway modification and therapeutic targeting. Since the discovery of the first miRNA twenty-six years ago, we have greatly increased our understanding of various genes’ functions and regulation within the cell. However, even though our foundation is solid, there are still gaping holes in our knowledge. There are numerous opportunities for progress to be made, particularly in the lesser-studied noncoding RNAs such as PIWI-RNAs and small nucleolar RNAs. In addition, the only well covered small RNA group in terms of the available animal models is the miRNAs. In conclusion, the roles noncoding RNAs play in the regulation of cellular apoptosis, activation, and regeneration in the progression of liver cancer, and the animal models used to study and elucidate these pathways, provide vast information for the potential development of therapeutic targets of hepatocellular carcinoma.

## Figures and Tables

**Table 1:** Summary of noncoding RNAs and the corresponding experimental animal models.

Animal Model	Type	Noncoding RNA	References
Xenograft	Nude	microRNA	[21,22,23,24]
		small interfering RNA	[25]
		small nucleolar RNA	[26,27]
	SCID	microRNA	[28,29]
	NOC-SCID	long noncoding RNA	[30,31]
Chemical	DEN	microRNA	[32,33,34,35,36]
		small interfering RNA	[37,38]
		long noncoding RNA	[39]
	CCl4	microRNA	[35,40,41]
		small interfering RNA	[37]
		long noncoding RNA	[42,43,44]
		PIWI RNA	[45]
Genetically Modified	HBx	microRNA	[46,47,48,49]
	HBsAg	microRNA	[46]
	MDR	microRNA	[50,51,52]
		long noncoding RNA	[53,54]
Other	Tet-o-myc	microRNA	[55]
	miR122 KO	microRNA	[56,57]
	miR148a deficient	microRNA	[36]
	KLF+/+, +/−	small interfering RNA	[37]
	Malat1 KO	small interfering RNA	[38]
	HCV-infected	microRNA	[58]
	miRNA nanoparticles	microRNA	[59]
	PH mouse	PIWI RNA	[45]
	PH bamboo shark	PIWI RNA	[60]

## References

[1] Anastasiadou E., Jacob L.S., Slack F.J. (2018). Non-coding RNA networks in cancer. Nat. Rev. Cancer.

[2] He Y., Meng X.-M., Huang C., Wu B.-M., Zhang L., Lv X.-W., Li J. (2014). Long noncoding RNAs: Novel insights into hepatocelluar carcinoma. Cancer Lett..

[3] Lee R.C., Feinbaum R.L., Ambros V. (1993). The C. elegans heterochronic gene lin-4 encodes small RNAs with antisense complementarity to lin-14. Cell.

[4] Wightman B., Ha I., Ruvkun G. (1993). Posttranscriptional regulation of the heterochronic gene lin-14 by lin-4 mediates temporal pattern formation in *C. elegans*. Cell.

[5] Carthew R.W., Sontheimer E.J. (2009). Origins and Mechanisms of miRNAs and siRNAs. Cell.

[6] Aravin A.A., Lagos-Quintana M., Yalcin A., Zavolan M., Marks D., Snyder B., Gaasterland T., Meyer J., Tuschl T. (2003). The Small RNA Profile during Drosophila melanogaster Development. Dev. Cell.

[7] Siomi H., Siomi M.C. (2009). On the road to reading the RNA-interference code. Nature.

[8] Kim V.N. (2006). Small RNAs just got bigger: Piwi-interacting RNAs (piRNAs) in mammalian testes. Genes Dev..

[9] Brennecke J., Aravin A.A., Stark A., Dus M., Kellis M., Sachidanandam R., Hannon G.J. (2007). Discrete Small RNA-Generating Loci as Master Regulators of Transposon Activity in Drosophila. Cell.

[10] Lim S.L., Tsend-Ayush E., Kortschak R.D., Jacob R., Ricciardelli C., Oehler M.K., Grützner F. (2013). Conservation and Expression of PIWI-Interacting RNA Pathway Genes in Male and Female Adult Gonad of *Amniotes 1*. Biol. Reprod..

[11] Chirn G.-W., Rahman R., Sytnikova Y.A., Matts J.A., Zeng M., Gerlach D., Yu M., Berger B., Naramura M., Kile B.T. (2015). Conserved piRNA Expression from a Distinct Set of piRNA Cluster Loci in Eutherian Mammals. PLoS Genet..

[12] Taft R.J., Glazov E.A., Lassmann T., Hayashizaki Y., Carninci P., Mattick J.S. (2009). Small RNAs derived from snoRNAs. RNA.

[13] Ender C., Krek A., Friedländer M.R., Beitzinger M., Weinmann L., Chen W., Pfeffer S., Rajewsky N., Meister G. (2008). A Human snoRNA with MicroRNA-Like Functions. Mol. Cell.

[14] Mitra J.C.a.S. (2018). Cancer and Noncoding RNAs.

[15] Iyer M.K., Niknafs Y.S., Malik R., Singhal U., Sahu A., Hosono Y., Barrette T.R., Prensner J.R., Evans J.R., Zhao S. (2015). The landscape of long noncoding RNAs in the human transcriptome. Nat. Genet..

[16] Derrien T., Johnson R., Bussotti G., Tanzer A., Djebali S., Tilgner H., Guernec G., Martin D., Merkel A., Knowles D.G. (2012). The GENCODE v7 catalog of human long noncoding RNAs: Analysis of their gene structure, evolution, and expression. Genome Res..

[17] Cheng C.-M., Shiah S.-G., Huang C.-C., Hsiao J.-R., Chang J.-Y. (2016). Up-regulation of miR-455-5p by the TGF-β–SMAD signalling axis promotes the proliferation of oral squamous cancer cells by targeting UBE2B. J. Pathol..

[18] Chen Z., Liu H., Jin W., Ding Z., Zheng S., Yu Y. (2016). Tissue microRNA-21 expression predicted recurrence and poor survival in patients with colorectal cancer—A meta-analysis. Onco Targets Ther..

[19] Feng J., Bi C., Clark B.S., Mady R., Shah P., Kohtz J.D. (2006). The Evf-2 noncoding RNA is transcribed from the Dlx-5/6 ultraconserved region and functions as a Dlx-2 transcriptional coactivator. Genes Dev..

[20] Tan X., Peng J., Fu Y., An S., Rezaei K., Tabbara S., Teal C.B., Man Y.G., Brem R.F., Fu S.W. (2014). miR-638 mediated regulation of BRCA1 affects DNA repair and sensitivity to UV and cisplatin in triple-negative breast cancer. Breast Cancer Res..

[21] Borel F., Konstantinova P., Jansen P.L. (2012). Diagnostic and therapeutic potential of miRNA signatures in patients with hepatocellular carcinoma. J. Hepatol..

[22] He L., Tian D.A., Li P.Y., He X.X. (2015). Mouse models of liver cancer: Progress and recommendations. Oncotarget.

[23] Zheng F., Liao Y.J., Cai M.Y., Liu Y.H., Liu T.H., Chen S.P., Bian X.W., Guan X.Y., Lin M.C., Zeng Y.X. (2012). The putative tumour suppressor microRNA-124 modulates hepatocellular carcinoma cell aggressiveness by repressing ROCK2 and EZH2. Gut.

[24] Meng F., Glaser S.S., Francis H., DeMorrow S., Han Y., Passarini J.D., Stokes A., Cleary J.P., Liu X., Venter J. (2012). Functional analysis of microRNAs in human hepatocellular cancer stem cells. J. Cell Mol. Med..

[25] Cheng L., Zhu Y., Han H., Zhang Q., Cui K., Shen H., Zhang J., Yan J., Prochownik E., Li Y. (2017). MicroRNA-148a deficiency promotes hepatic lipid metabolism and hepatocarcinogenesis in mice. Cell Death Dis..

[26] Lai M.C., Yang Z., Zhou L., Zhu Q.Q., Xie H.Y., Zhang F., Wu L.M., Chen L.M., Zheng S.S. (2012). Long non-coding RNA MALAT-1 overexpression predicts tumor recurrence of hepatocellular carcinoma after liver transplantation. MO.

[27] Miard S., Girard M.J., Joubert P., Carter S., Boivin L., Picard F., Gonzales A., Guo H., Morpurgo B., Golovko A. (2017). Absence of Malat1 does not prevent DEN-induced hepatocarcinoma in mice. Oncol. Rep..

[28] Ding J., Huang S., Wu S., Zhao Y., Liang L., Yan M., Ge C., Yao J., Chen T., Wan D. (2010). Gain of miR-151 on chromosome 8q24.3 facilitates tumour cell migration and spreading through downregulating RhoGDIA. Nat. Cell Biol..

[29] Wong C.C., Wong C.M., Tung E.K., Au S.L., Lee J.M., Poon R.T., Man K., Ng I.O. (2011). The microRNA miR-139 suppresses metastasis and progression of hepatocellular carcinoma by down-regulating Rho-kinase 2. Gastroenterology.

[30] Tarocchi M., Hannivoort R., Hoshida Y., Lee U.E., Vetter D., Narla G., Villanueva A., Oren M., Llovet J.M., Friedman S.L. (2011). Carcinogen-induced hepatic tumors in KLF6+/− mice recapitulate aggressive human hepatocellular carcinoma associated with p53 pathway deregulation. Hepatology.

[31] Ozata D.M., Gainetdinov I., Zoch A., O’Carroll D., Zamore P.D. (2019). PIWI-interacting RNAs: Small RNAs with big functions. Nat. Rev. Genet..

[32] Ma S., Tang K.H., Chan Y.P., Lee T.K., Kwan P.S., Castilho A., Ng I., Man K., Wong N., To K.F. (2010). miR-130b Promotes CD133(+) liver tumor-initiating cell growth and self-renewal via tumor protein 53-induced nuclear protein 1. Cell Stem Cell.

[33] Lampis A., Carotenuto P., Vlachogiannis G., Cascione L., Hedayat S., Burke R., Clarke P., Bosma E., Simbolo M., Scarpa A. (2018). MIR21 Drives Resistance to Heat Shock Protein 90 Inhibition in Cholangiocarcinoma. Gastroenterology.

[34] Buchmann A., Bauer-Hofmann R., Mahr J., Drinkwater N.R., Luz A., Schwarz M. (1991). Mutational activation of the c-Ha-ras gene in liver tumors of different rodent strains: Correlation with susceptibility to hepatocarcinogenesis. Proc. Natl. Acad. Sci. USA.

[35] Wu K., Ding J., Chen C., Sun W., Ning B.F., Wen W., Huang L., Han T., Yang W., Wang C. (2012). Hepatic transforming growth factor beta gives rise to tumor-initiating cells and promotes liver cancer development. Hepatology.

[36] Kota J., Chivukula R.R., O’Donnell K.A., Wentzel E.A., Montgomery C.L., Hwang H.W., Chang T.C., Vivekanandan P., Torbenson M., Clark K.R. (2009). Therapeutic microRNA delivery suppresses tumorigenesis in a murine liver cancer model. Cell.

[37] Di Ianni T., Bose R.J.C., Sukumar U.K., Bachawal S., Wang H., Telichko A., Herickhoff C., Robinson E., Baker S., Vilches-Moure J.G. (2019). Ultrasound/microbubble-mediated targeted delivery of anticancer microRNA-loaded nanoparticles to deep tissues in pigs. J. Control. Release.

[38] Yan X., Chua M.-S., He J., So S.K. (2008). Small interfering RNA targeting CDC25B inhibits liver tumor growth in vitro and in vivo. Mol. Cancer.

[39] Fang X., Yang D., Luo H., Wu S., Dong W., Xiao J., Yuan S., Ni A., Zhang K.-J., Liu X.-Y. (2017). SNORD126 promotes HCC and CRC cell growth by activating the PI3K-AKT pathway through FGFR2. J. Mol. Cell Biol..

[40] Fornari F., Pollutri D., Patrizi C., La Bella T., Marinelli S., Casadei Gardini A., Marisi G., Baron Toaldo M., Baglioni M., Salvatore V. (2017). In Hepatocellular Carcinoma miR-221 Modulates Sorafenib Resistance through Inhibition of Caspase-3-Mediated Apoptosis. Clin. Cancer Res..

[41] Callegari E., Elamin B.K., Giannone F., Milazzo M., Altavilla G., Fornari F., Giacomelli L., D’Abundo L., Ferracin M., Bassi C. (2012). Liver tumorigenicity promoted by microRNA-221 in a mouse transgenic model. Hepatology.

[42] Tang Q., Wang Q., Zhang Q., Lin S.-Y., Zhu Y., Yang X., Guo A.-Y. (2017). Gene expression, regulation of DEN and HBx induced HCC mice models and comparisons of tumor, para-tumor and normal tissues. BMC Cancer.

[43] Rizzo F., Hashim A., Marchese G., Ravo M., Tarallo R., Nassa G., Giurato G., Rinaldi A., Cordella A., Persico M. (2014). Timed regulation of P-element-induced wimpy testis–interacting RNA expression during rat liver regeneration. Hepatology.

[44] Yang L., Ge Y., Cheng D., Nie Z., Lv Z. (2016). Detection of piRNAs in whitespotted bamboo shark liver. Gene.

[45] Chu P., Wang Q., Wang Z., Gao C. (2019). Long non-coding RNA highly up-regulated in liver cancer protects tumor necrosis factor-alpha-induced inflammatory injury by down-regulation of microRNA-101 in ATDC5 cells. Int. Immunopharmacol..

[46] Callegari E., Domenicali M., Shankaraiah R.C., D’Abundo L., Guerriero P., Giannone F., Baldassarre M., Bassi C., Elamin B.K., Zagatti B. (2019). MicroRNA-Based Prophylaxis in a Mouse Model of Cirrhosis and Liver Cancer. Mol. Ther. Nucleic Acids.

[47] Hyun J., Wang S., Kim J., Rao K.M., Park S.Y., Chung I., Ha C.S., Kim S.W., Yun Y.H., Jung Y. (2016). MicroRNA-378 limits activation of hepatic stellate cells and liver fibrosis by suppressing Gli3 expression. Nat. Commun..

[48] Marrone A.K., Shpyleva S., Chappell G., Tryndyak V., Uehara T., Tsuchiya M., Beland F.A., Rusyn I., Pogribny I.P. (2016). Differentially expressed MicroRNAs provide mechanistic insight into fibrosis-associated liver carcinogenesis in mice. Mol. Carcinog..

[49] Wang Y., Cui F., Lv Y., Li C., Xu X., Deng C., Wang D., Sun Y., Hu G., Lang Z. (2004). HBsAg and HBx knocked into the p21 locus causes hepatocellular carcinoma in mice. Hepatology.

[50] Moriya K., Fujie H., Shintani Y., Yotsuyanagi H., Tsutsumi T., Ishibashi K., Matsuura Y., Kimura S., Miyamura T., Koike K. (1998). The core protein of hepatitis C virus induces hepatocellular carcinoma in transgenic mice. Nat. Med..

[51] Zhang Y., Wei W., Cheng N., Wang K., Li B., Jiang X., Sun S. (2012). Hepatitis C virus-induced up-regulation of microRNA-155 promotes hepatocarcinogenesis by activating Wnt signaling. Hepatology.

[52] Katzenellenbogen M., Pappo O., Barash H., Klopstock N., Mizrahi L., Olam D., Jacob-Hirsch J., Amariglio N., Rechavi G., Mitchell L.A. (2006). Multiple adaptive mechanisms to chronic liver disease revealed at early stages of liver carcinogenesis in the Mdr2-knockout mice. Cancer Res..

[53] Zhu P., Wang Y., Wu J., Huang G., Liu B., Ye B., Du Y., Gao G., Tian Y., He L. (2016). LncBRM initiates YAP1 signalling activation to drive self-renewal of liver cancer stem cells. Nat. Commun..

[54] Tang S.-H., Gao J.-H., Wen S.-L., Tong H., Yan Z.-P., Liu R., Tang C.-W. (2017). Expression of cyclooxygenase-2 is correlated with lncRNA-COX-2 in cirrhotic mice induced by carbon tetrachloride. Mol. Med. Rep..

[55] Wu N., Meng F., Zhou T., Han Y., Kennedy L., Venter J., Francis H., DeMorrow S., Onori P., Invernizzi P. (2017). Prolonged darkness reduces liver fibrosis in a mouse model of primary sclerosing cholangitis by miR-200b down-regulation. Faseb J..

[56] Sato K., Meng F., Glaser S., Alpini G. (2016). Exosomes in liver pathology. J. Hepatol..

[57] McDaniel K., Wu N., Zhou T., Huang L., Sato K., Venter J., Ceci L., Chen D., Ramos-Lorenzo S., Invernizzi P. (2019). Amelioration of Ductular Reaction by Stem Cell Derived Extracellular Vesicles in MDR2 Knockout Mice via Lethal-7 microRNA. Hepatology.

[58] Huang J., Wang Y., Guo Y., Sun S. (2010). Down-regulated microRNA-152 induces aberrant DNA methylation in hepatitis B virus-related hepatocellular carcinoma by targeting DNA methyltransferase 1. Hepatology.

[59] Hsu S.H., Wang B., Kota J., Yu J., Costinean S., Kutay H., Yu L., Bai S., La Perle K., Chivukula R.R. (2012). Essential metabolic, anti-inflammatory, and anti-tumorigenic functions of miR-122 in liver. J. Clin. Invest..

[60] Lafontaine D.L.J., Tollervey D. (1998). Birth of the snoRNPs: The evolution of the modification-guide snoRNAs. Trends Biochem. Sci..

[61] Alles J., Fehlmann T., Fischer U., Backes C., Galata V., Minet M., Hart M., Abu-Halima M., Grasser F.A., Lenhof H.P. (2019). An estimate of the total number of true human miRNAs. Nucleic Acids Res..

[62] Friedman R.C., Farh K.K., Burge C.B., Bartel D.P. (2009). Most mammalian mRNAs are conserved targets of microRNAs. Genome Res..

[63] Wong C.M., Tsang F.H., Ng I.O. (2018). Non-coding RNAs in hepatocellular carcinoma: Molecular functions and pathological implications. Nat. Rev. Gastroenterol. Hepatol..

[64] Kim Y.K., Kim B., Kim V.N. (2016). Re-evaluation of the roles of DROSHA, Export in 5, and DICER in microRNA biogenesis. Proc. Natl. Acad. Sci. USA.

[65] Ha M., Kim V.N. (2014). Regulation of microRNA biogenesis. Nat. Rev. Mol. Cell Biol..

[66] Huntzinger E., Izaurralde E. (2011). Gene silencing by microRNAs: Contributions of translational repression and mRNA decay. Nat. Rev. Genet..

[67] Cui X.W., Qian Z.L., Li C., Cui S.C. (2018). Identification of miRNA and mRNA expression profiles by PCR microarray in hepatitis B virusassociated hepatocellular carcinoma. Mol. Med. Rep..

[68] Roy S., Hooiveld G.J., Seehawer M., Caruso S., Heinzmann F., Schneider A.T., Frank A.K., Cardenas D.V., Sonntag R., Luedde M. (2018). microRNA 193a-5p Regulates Levels of Nucleolar- and Spindle-Associated Protein 1 to Suppress Hepatocarcinogenesis. Gastroenterology.

[69] Teufel M., Seidel H., Kochert K., Meinhardt G., Finn R.S., Llovet J.M., Bruix J. (2019). Biomarkers Associated With Response to Regorafenib in Patients With Hepatocellular Carcinoma. Gastroenterology.

[70] Wong C.M., Wei L., Au S.L., Fan D.N., Zhou Y., Tsang F.H., Law C.T., Lee J.M., He X., Shi J. (2015). MiR-200b/200c/429 subfamily negatively regulates Rho/ROCK signaling pathway to suppress hepatocellular carcinoma metastasis. Oncotarget.

[71] Banales J.M., Cardinale V., Carpino G., Marzioni M., Andersen J.B., Invernizzi P., Lind G.E., Folseraas T., Forbes S.J., Fouassier L. (2016). Expert consensus document: Cholangiocarcinoma: Current knowledge and future perspectives consensus statement from the European Network for the Study of Cholangiocarcinoma (ENS-CCA). Nat. Rev. Gastroenterol. Hepatol..

[72] Han Y., Meng F., Venter J., Wu N., Wan Y., Standeford H., Francis H., Meininger C., Greene J., Trzeciakowski J.P. (2016). miR-34a-dependent overexpression of Per1 decreases cholangiocarcinoma growth. J. Hepatol..

[73] Ehrlich L., Hall C., Venter J., Dostal D., Bernuzzi F., Invernizzi P., Meng F., Trzeciakowski J.P., Zhou T., Standeford H. (2017). miR-24 Inhibition Increases Menin Expression and Decreases Cholangiocarcinoma Proliferation. Am. J. Pathol..

[74] Lu Y.S., Kashida Y., Kulp S.K., Wang Y.C., Wang D., Hung J.H., Tang M., Lin Z.Z., Chen T.J., Cheng A.L. (2007). Efficacy of a novel histone deacetylase inhibitor in murine models of hepatocellular carcinoma. Hepatology.

[75] Zhang X., Liu S., Hu T., Liu S., He Y., Sun S. (2009). Up-regulated microRNA-143 transcribed by nuclear factor kappa B enhances hepatocarcinoma metastasis by repressing fibronectin expression. Hepatology.

[76] Lan S.H., Wu S.Y., Zuchini R., Lin X.Z., Su I.J., Tsai T.F., Lin Y.J., Wu C.T., Liu H.S. (2014). Autophagy suppresses tumorigenesis of hepatitis B virus-associated hepatocellular carcinoma through degradation of microRNA-224. Hepatology.

[77] Valdmanis P.N., Kim H.K., Chu K., Zhang F., Xu J., Munding E.M., Shen J., Kay M.A. (2018). miR-122 removal in the liver activates imprinted microRNAs and enables more effective microRNA-mediated gene repression. Nat. Commun..

[78] Zhang S., Nguyen L.H., Zhou K., Tu H.-C., Sehgal A., Nassour I., Li L., Gopal P., Goodman J., Singal A.G. (2018). Knockdown of Anillin Actin Binding Protein Blocks Cytokinesis in Hepatocytes and Reduces Liver Tumor Development in Mice Without Affecting Regeneration. Gastroenterology.

[79] Wu L., Zheng J., Chen P., Liu Q., Yuan Y. (2017). Small nucleolar RNA ACA11 promotes proliferation, migration and invasion in hepatocellular carcinoma by targeting the PI3K/AKT signaling pathway. Biomed. Pharmacother..

[80] Udager A.M., Smith S.C., Tomlins S.A., Coleman W.B., Tsongalis G.J. (2017). Chapter 22—Molecular Pathology of Prostate Cancer. Diagnostic Molecular Pathology.

[81] Bard-Chapeau E.A., Li S., Ding J., Zhang S.S., Zhu H.H., Princen F., Fang D.D., Han T., Bailly-Maitre B., Poli V. (2011). Ptpn11/Shp2 Acts as a Tumor Suppressor in Hepatocellular Carcinogenesis. Cancer Cell.

[82] Cui H., Zhang Y., Zhang Q., Chen W., Zhao H., Liang J. (2017). A comprehensive genome-wide analysis of long noncoding RNA expression profile in hepatocellular carcinoma. Cancer Med..

[83] Wang H.H., Guan Z.H., He K.F., Qian J., Cao J., Teng L.S. (2017). LncRNA UCA1 in anti-cancer drug resistance. Oncotarget.

[84] Sadek K.M., Lebda M.A., Nasr N.E., Nasr S.M., El-Sayed Y. (2018). Role of lncRNAs as prognostic markers of hepatic cancer and potential therapeutic targeting by S-adenosylmethionine via inhibiting PI3K/Akt signaling pathways. ESPR.

[85] Katzenellenbogen M., Mizrahi L., Pappo O., Klopstock N., Olam D., Jacob-Hirsch J., Amariglio N., Rechavi G., Domany E., Galun E. (2007). Molecular Mechanisms of Liver Carcinogenesis in the Mdr2-Knockout Mice. Mol. Cancer Res..

[86] Li X., Liu R., Yang J., Sun L., Zhang L., Jiang Z., Puri P., Gurley E.C., Lai G., Tang Y. (2017). The role of long noncoding RNA H19 in gender disparity of cholestatic liver injury in multidrug resistance 2 gene knockout mice. Hepatology..

